# NFIB overexpression cooperates with *Rb/p53* deletion to promote small cell lung cancer

**DOI:** 10.18632/oncotarget.11583

**Published:** 2016-08-24

**Authors:** Nan Wu, Deshui Jia, Ali H. Ibrahim, Cindy J. Bachurski, Richard M. Gronostajski, David MacPherson

**Affiliations:** ^1^ Divisions of Human Biology and Public Health Sciences, Fred Hutchinson Cancer Research Center, Seattle, WA, USA; ^2^ Division of Pulmonary Biology, Cincinnati Children's Hospital Research Foundation, Cincinnati, OH, USA; ^3^ Department of Biochemistry, Program in Genetics, Genomics & Bioinformatics, University at Buffalo, Buffalo, NY, USA; ^4^ Department of Genome Sciences, University of Washington, Seattle, WA, USA

**Keywords:** oncogene, metastasis, mouse model, nuclear factor I, NFI

## Abstract

Small cell lung cancer (SCLC) is a highly aggressive neuroendocrine tumor type that is typically metastatic upon diagnosis. We have a poor understanding of the factors that control SCLC progression and metastasis. TheNFIB transcription factor is frequently amplified in mouse models of SCLC, but clear evidence that *NFIB* promotes SCLC in vivo is lacking. We report that in mouse models, *Nfib* amplifications are far more frequent in liver metastases over primary SCLC, suggesting roles in tumor progression/metastasis. Overexpression of *Nfib* in a sensitized mouse model led to acceleration of SCLC, indicating that *Nfib* functions as a bona fide oncogene. Suppression of *Nfib* expression in cell lines derived from the doxycycline-inducible *Rb/p53/TET-Nfib* model led to increased apoptosis and suppression of proliferation. Transcriptional analysis revealed that *Nfib* regulates the expression of genes related to axon guidance, focal adhesion and extracellular matrix-receptor interactions. These data indicate that *Nfib* is a potent oncogene in SCLC, and the enrichment of *Nfib* amplifications in liver metastases over primary SCLC points to *Nfib* as a candidate driver of SCLC metastasis.

## INTRODUCTION

The most metastatic form of lung cancer is SCLC, a highly aggressive neuroendocrine cancer [1]. SCLC is typically metastatic at initial diagnosis, but little is known about the why this is so. The high frequency of metastasis at diagnosis raises the possibility that SCLC may acquire many of the genetic lesions needed for metastasis early, but there has been little work to compare primary SCLC to metastases using human samples. This is likely owing to the difficulty in accessing samples from a tumor type that rarely undergoes surgical resection. Thus, the timing of both genetic and non-genetic changes that promote SCLC progression and metastasis are very poorly understood.

Study of mouse models can overcome the limitations in accessing SCLC clinical samples. Human SCLCs almost universally exhibit *RB* and *P53* inactivation [2, 3] and *Rb/p53* deletion in a subset of cells in the murine lung leads to SCLC that arises with near complete penetrance [4, 5]. The resulting tumors resemble human SCLC, exhibiting strong neuroendocrine characteristics and frequent metastasis, especially to the liver. Tumors in the *Rb/p53* mouse model undergo spontaneous secondary genetic alterations that also occur in human SCLC such as amplification of *Mycl* or deletion of *Pten* [6]. Moreover, overexpression of *Mycl* [7, 8] or deletion of *Pten* [6, 9] accelerates murine SCLC, showing that mouse models can be used to interrogate potential SCLC driver genes. We need a better understanding of which genetic changes occur very early in tumor initiation and which subsequent mutations contribute to tumor progression and metastasis.

Beyond *Mycl* amplification and *Pten* deletion, another genetic alteration that occurs in mouse models of SCLC is focal amplification of the *Nfib* gene [10]. These amplifications are high-level, and *Nfib* was found to be the only gene in the minimal region of amplification [10]. *Nfib* is one of four members of the *Nfi* family of transcription factors, a family that includes *Nfia, Nfib, Nfic,* and *Nfix.* Genetic inactivation of *Nfib* in mice revealed that *Nfib* is important both for brain and lung development [11, 12]. In human SCLC cells, *NFIB* can sometimes undergo high-level amplification [10]. An alternative mechanism through which *NFIB* can be highly expressed is through the presence of atypically large transcriptional enhancers or super-enhancers [13, 14]. Super-enhancers, marked by high levels and long stretches of histone H3K27 acetylation (and/or other features associated with active enhancers) have been proposed to promote the expression of genes important for cell lineage and oncogenesis [15–17]. Cell based studies support the notion that *NFIB* exhibits oncogenic activity [10], but *in vivo* analyses are required to rigorously assess *Nfib* oncogenic function. In this study, we overexpressed *Nfib* in a sensitized mouse model and also suppressed NFIB expression in SCLC cells; we report potent oncogenic activity of *Nfib* in promoting SCLC *in vivo*.

## RESULTS

### Nfib amplifications occur more frequently in liver metastases over primary SCLC

**Figure 1:**
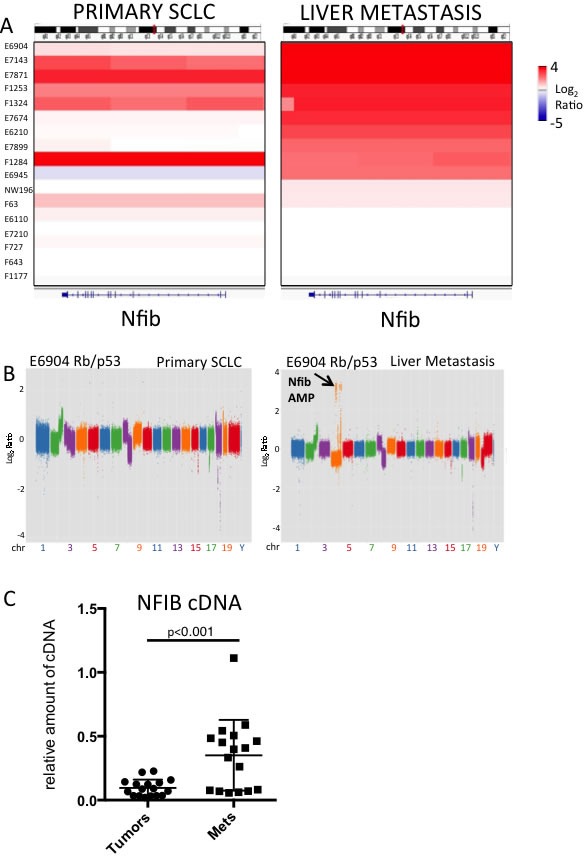
*Nfib* is amplified in liver metastases over primary tumors in mouse models of SCLC **A.** Integrated genome viewer (IGV) plot showing *Nfib* copy number in a series of 17 primary murine SCLCs (left) and liver metastases (right). Log_2_ copy number ratios of tumor DNA relative to normal tail DNA is shown, with scale to the right. **B.** Copy number variation (CNV) analysis showing matched pair of primary SCLC and liver metastases, analyzed using CNVSeq. The majority of copy number alterations are similar between the primary and metastasis. Chromosome 4 harbors focal amplifications including *Nfib* amplification (arrow) exclusively in the metastasis. **C.** Real-time PCR analysis of *Nfib* expression (bottom) across a panel of primary SCLC and liver metastases. Data are normalized to *Gapdh*. Note the clear upregulation in *Nfib* expression in a large proportion of the liver metastases over matched primary lung tumors. p-value from Student's T-test is indicated.

We used next generation sequencing to compare genetic alterations between primary SCLC and liver metastases that arise in murine models of SCLC. These models are initiated by intra-tracheal delivery of either broadly expressing Ad-CMV-Cre or Ad-Cre driven by a neuroendocrine promoter (Ad-CGRP-Cre). We collected primary tumors and liver metastases across a series of 17 mice derived from three models: *Rb^lox/lox^;p53^lox/lox^* [4] and *Rb^lox/lox^;p53^lox/lox^;Pten^lox/+^* [9] mice infected with Ad-CMV-Cre, and an *Rb^lox/lox^;p53^lox/lox^;Pten^lox/lox^* model infected with Ad-CGRP-Cre [6, 7]. The use of neuroendocrine specific Ad-CGRP-Cre is necessary in the *Rb^lox/lox^;p53^lox/lox^;Pten^lox/lox^* model, as this dramatically reduces the incidence of adenocarcinoma that otherwise occurs with Ad-CMV-Cre infection in these mice [6, 7, 9]. We analyzed one lung tumor and one liver metastasis from each of 17 SCLC-bearing mice (Supplemental Table 1). We performed copy number variation (CNV) analyses using low-coverage whole genome sequencing across each lung tumor and liver metastasis. Matched normal tail DNA was used as a control. We were particularly interested in recurrent changes enriched in metastatic tumors. We employed Segseq [18] and CNVseq [19] to identify CNV changes. We did not find recurrent examples of metastasis-specific enrichment or newly acquired genetic amplifications in *Mycl* or deletions of *Pten* in the liver metastases (Supplemental Figure 1A, 2A and data not shown). However, we identified a region of focal amplification on chromosome 4 that was very frequently amplified in liver metastases over primary SCLC, this region harbored the *Nfib* gene (Figure 1A, Supplemental Figure 2). In the mouse models tested, multiple primary tumors can arise in the lung and so the liver metastasis was not always clonally related to the paired lung tumor. However, across some of the pairs, obvious clonal relationships could be discerned where it was clear that the *Nfib* amplification was present in the liver metastasis but undetectable in the bulk primary lung tumor. For example, Figure 1B shows a tumor from the *Rb/p53* model, H6904, with very similar genome-wide CNV profiles between the lung and liver metastasis. However, multiple chromosome 4 amplifications, including *Nfib* (but not *Mycl*) were present in the liver metastasis but not the matched lung tumor. Similarly, across another *Rb/p53* model pair that was clearly clonally related (H6210), *Mycl* amplification was present in the lung SCLC (which lacked *Nfib* amplification), and the liver metastasis exhibited *Nfib* amplification but lacked *Mycl* amplification (Supplemental Figure 1B). This example suggests different evolution of the lung tumor and liver metastasis by the time of sampling. We also compared *Nfib* expression across a series of primary SCLC *vs*. metastases from the *Rb/p53* and *Rb/p53/Pten^lox/+^* models using real time PCR. We found significantly increased expression of *Nfib* in liver metastases over primary tumors (Figure 1C). We conclude that *Nfib* amplifications are enriched in liver metastases over primary tumors in mouse SCLC models. Our data suggest that positive selection of SCLC cells harboring *Nfib* amplification may contribute to SCLC progression.

### Nfib overexpression accelerates SCLC in a mouse model

**Figure 2:**
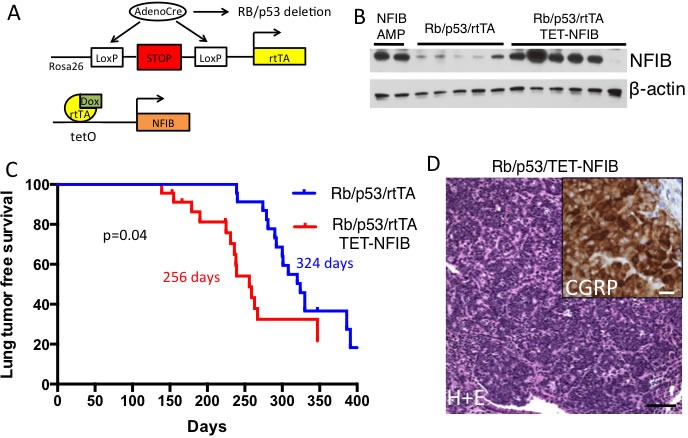
*Nfib* overexpression accelerates SCLC *in vivo* **A.** Cartoon showing doxycycline inducible *Nfib* expression and *Rb/p53* deletion driven by Adeno *Cre* expression, delivered using intratracheal delivery to the lung epithelium. **B.** Western blot showing NFIB overexpression in lung tumors from the *Rb/p53/TET-Nfib* model, as compared to *Rb/p53* model controls. As a positive control, two liver metastases from tumors harboring spontaneous genomic amplification of *Nfib* in the *Rb/p53* model are also shown (NFIB AMP). **C.** Kaplan-Meier curve showing acceleration of SCLC in *Rb/p53/TET-Nfib* compared to the *Rb/p53* model. Median survival for each model is indicated and p-value from log-rank test is shown. **D.** Hematoxylin and eosin stain of representative SCLC arising in the *Rb/p53/TET-Nfib* model, black scale bar, 50mm. Inset: CGRP immunostaining of *Rb/p53/TET-Nfib* tumor, white scale bar, 10mm.

We next used a novel TET-regulated *Nfib* transgenic strain (*TRE-Nfib*) to investigate the role of *Nfib* in SCLC initiation (see Methods for details on transgenic strain generation). By combining this allele with the *Rosa26^lox-stop-lox rtTA^* allele, *rtTA,* and consequently *Nfib,* is overexpressed upon Cre-mediated recombination (Figure 2A). Intra-tracheal AdenoCre delivery leads to Cre expression in lung epithelial cells. We used this inducible system to test oncogenic roles for *Nfib* in the sensitized *Rb^lox/lox^;p53^lox/lox^* AdenoCre SCLC model [4]. We compared tumorigenesis in *Rb^lox/lox^;p53^lox/lox^;Rosa26^LSL-rtTA/LSL-rtTA^;TRE-Nfib* (herein referred to as *Rb/p53/TET-Nfib*) *vs*. *Rb^lox/lox^;p53^lox/lox^;Rosa26^LSL-rtTA/LSL-rtTA^* mice (herein referred to as *Rb/p53*). The expression of *Nfib* is activated by addition of doxycycline to the feed starting one week after Ad-CMV-Cre infection. We followed mice until respiratory changes from tumor burden necessitated euthanasia. Western blot analysis of lung tumors collected from *Rb/p53/TET-Nfib* mice showed increased expression of NFIB compared to lung tumors from *Rb/p53* controls (Figure 2B). As shown in Figure 2C, we found that *Nfib* overexpression rapidly accelerated time to SCLC with a median time to morbidity of 256 days in the *Rb/p53/TET-Nfib* model *vs*. 324 days in the *Rb/p53* controls (*p* = 0.04, log-rank test). Resulting tumors in the *Nfib* overexpressing model exhibited histology typical of the *Rb/p53* model [20] and exhibited neuroendocrine features, as shown by Calcitonin Gene Related Peptide (CGRP) immunostaining (Figure 2D). While *Rb/p53* deletion in this system leads to predominant SCLC, cooperating mutations can lead to a change in tumor spectrum, as in Cui et al [9], where *Pten* inactivation promoted lung adenocarcinoma. We found no instances of adenocarcinoma in the *Rb/p53/TET-Nfib* group. Given *Nfib* amplification enrichment in liver metastases (Figure 1), we were interested in whether *Nfib*-overexpressing SCLC exhibited higher rates of liver metastasis. Upon necropsy, gross liver metastases were found in 6/14 (43%) of lung tumor bearing mice from the *Rb/p53/TET-Nfib* compared to 7/15 (47%) of lung tumor bearing mice from the *Rb/p53* group. Liver metastases were found at an average+/−s.d. of 299+/−53 days in the *Rb/p53/TET-Nfib* model and 318+/−29 days in the *Rb/p53* model, a difference that was not statistically significant. Our data indicate that *Nfib* functions as a potent lung cancer oncogene in the *Rb/p53* model and this ability of *Nfib* to function as an oncogene was not specific to SCLC metastasis.

### Suppressing NFIB levels reduces proliferation

**Figure 3:**
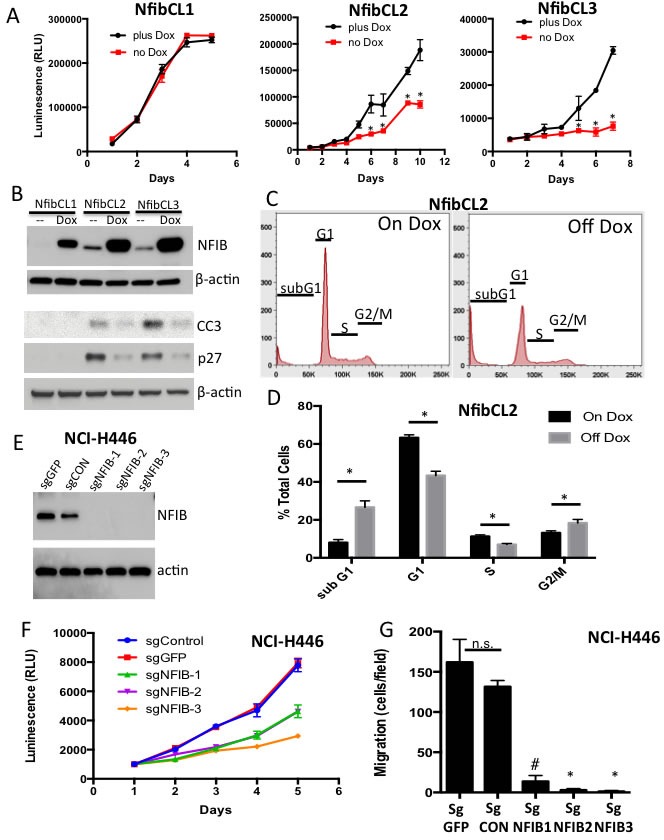
Suppression of NFIB expression reduces cell viability and migration **A.** Cell TiterGlo assay showing proliferation in *Rb/p53/TET-Nfib* cell lines upon removal of doxycycline (DOX). Mean +/− s.d. of triplicate wells shown and data are representative of 3 independent experiments. *p < 0.001, Student's T-test. **B.** Western blot showing NFIB, cleaved Caspase 3 (CC3) and p27 protein levels across three *Rb/p53/TET-Nfib* cell lines in the presence of DOX or 7 days post washout of DOX. Beta actin is used as a loading control. **C.** FACS analysis for DNA content shows increased subG1 population, indicative of apoptosis, and decreased G1 and S-phase populations upon doxycycline removal in NfibCL2 cell line from *Rb/p53/TET-Nfib* lung tumor. **D.** Quantification of FACS data. Data representative of 2 independent experiments, each done in triplicate. *p < 0.001, Student's T-test. **E.** Western blot showing NFIB expression in NCI-H446 cell line that underwent lentiviral CRISPR-mediated *NFIB* deletion. Three *NFIB*-deleted sub-lines, derived from single cells, are shown along with two control sublines with guide RNAs targeting GFP, or a non-targeting control guide RNA. Two different sgRNA sequences were used, with one clone using sgRNA sequence NFIB#1 and two clones using sgRNA sequence NFIB#2 (see methods). **F.** CellTiter Glo experiment showing reduced proliferation in the *NFIB-*deleted H446 cells. **G.** Transwell migration assay in which *NFIB*-deleted and control H446 cells were transferred to transwells and allowed to migrate over 17 hours. Crystal violet stained migrated cells were counted. Each cell line was plated in triplicate transwells and results show mean +/− s.d. * *p* < 0.001, # *p* < 0.005, Student's T-test.

We established cell lines from *Rb/p53/TET-Nfib* mice that had *Nfib* overexpressed in a doxycycline-dependent fashion. By washing out doxycycline from the media after cell line establishment, we could interrogate the cellular consequences of suppressing NFIB expression in tumors initiated with high levels of NFIB. Upon removal of doxycycline from the media, we found suppression of proliferation in two of three cell lines (Figure 3A) and sharply decreased NFIB protein levels (Figure 3B). In the responsive cell lines, removal of doxycycline led to increased apoptosis. We observed an increased sub-G1 population in FACS analysis for DNA content (Figure 3C, 3D and not shown). Increased cleaved caspase 3 was consistent with higher levels of apoptosis in responsive cells upon doxycycline removal (Figure 3B). We also found a decrease in the proportion of cells in S-phase upon NFIB suppression (Figure 3C, 3D, and data not shown), which correlated with increased expression of the cyclin dependent kinase inhibitor p27 in Western blot analysis (Figure 3B). In a human SCLC cell line, NCI-H446 that harbors a focal *NFIB* amplification [10] we used lentiviral CRISPR to generate *NFIB*-deleted single cell clones and confirmed complete loss of NFIB expression by Western blot analysis (Figure 3E). Deletion of *NFIB* across independent clones resulted in decreased numbers of viable cells (Figure 3F) and reduced cell migration, as assessed using a transwell migration assay (Figure 3G). Thus, NFIB regulates cell viability and migration in SCLC cells.

### NFIB transcriptional programs in SCLC

**Figure 4:**
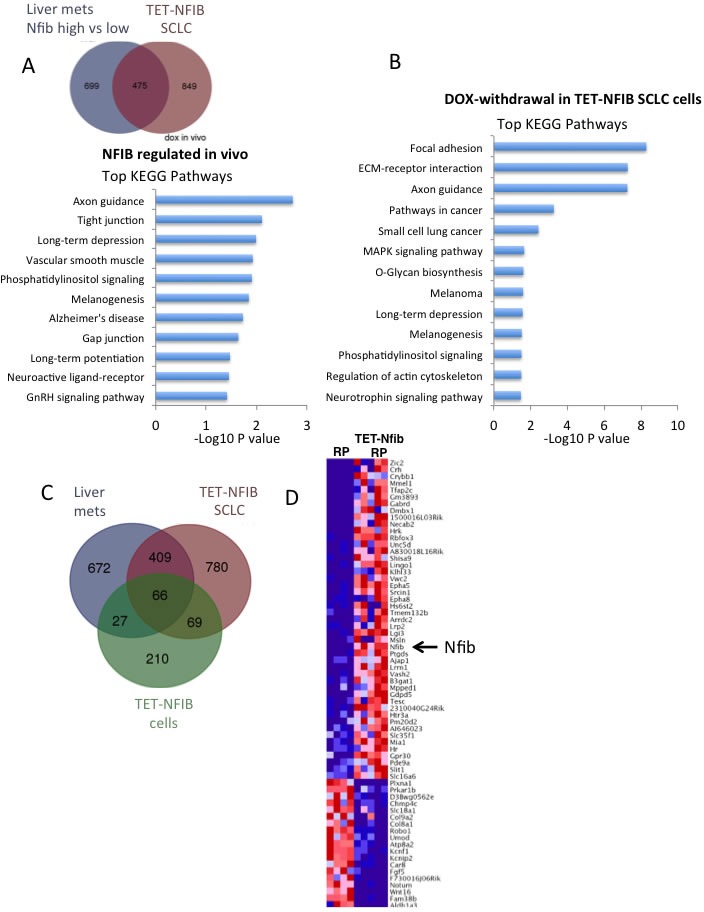
NFIB controlled transcriptional programs in SCLC **A.** Top: Venn diagram showing overlapping differential expressed genes across two comparisons, including liver metastases with high *vs*. low *Nfib* expression and lung SCLCs from the *Rb/p53* vs. *Rb/p53/TET-Nfib* models. 475 overlapping genes were differentially expressed in both liver metastasis and DOX-inducible lung tumor comparisons. Bottom: Top KEGG pathways enriched in this gene set are indicated with *p*-value shown on X-axis. **B.** KEGG pathways enriched in 372 genes differentially expressed in an RNAseq comparison of 4 *Rb/p53/TET-Nfib* SCLC cell lines, comparing cells on doxycycline *vs*. 7 days after doxycycline washout **C.** Venn diagram showing 66 genes were commonly differentially expressed across all three comparisons. **D.** Heat map showing the expression of the commonly altered 66 genes in the comparison of lung SCLCs from the Rb/p53 *vs*. Rb/p53/TET-Nfib models.

As a transcription factor that promotes SCLC, we were interested in identifying NFIB-regulated transcriptional programs in SCLC cells. The SCLC tumors and cells that differ in NFIB expression generated in this study provided ideal reagents to identify NFIB-regulated pathways. We performed RNAseq analyses on three complementary datasets. First, we compared gene expression in liver metastases from the *Rb/p53* and *Rb/p53/Pten^lox/+^* models that exhibited high *vs*. low *Nfib* expression. Using RNAseq and EdgeR analysis, we identified 1174 genes to be differentially expressed (FDR <0.05) (Supplemental Table 2). Second, we compared SCLCs from the *Rb/p53 (RTTA* controls) *vs*. *Rb/p53/TET-Nfib* lung tumors that arose in the presence of doxycycline. Here, we identified 1324 genes that were differentially expressed in an NFIB dependent manner (Supplemental Table 3); 475 of these overlapped with the differentially expressed genes from the previous liver metastasis comparison (Figure 4A). Kyoto Encyclopedia of Genes and Genomes (KEGG) pathway analysis of this 475-gene set using DAVID (Database for Annotation, Visualization and Integrated Discovery) revealed significant enrichment in axon guidance, tight junctions, phosphatidylinositol signaling, and other pathways (Figure 4A). Third, we compared SCLC cell lines derived from *Rb/p53/TET-Nfib* tumors that were maintained in the presence of DOX or collected 7 days following DOX removal; here we identified 372 differentially expressed genes (Supplemental Table 4). This comparison was particularly important, as we expect that all of the cells are tumor cells, in contrast to the analyses of tumor tissues, where gene expression changes include a stromal cell contribution. KEGG analysis of this 372-gene set revealed highly significant NFIB-dependent pathways (focal adhesion *p* = 5*10^−9^, extracellular matrix-receptor interaction *p* = 5*10^−8^, axon guidance, *p* = 5*10^−8^ and pathways in cancer *p* = 5*10^−4^) shown in Figure 4B. Considering all three differential expression comparisons, i.e. liver metastases with high *vs*. low *Nfib* expression, DOX-inducible *Rb/p53/TET-Nfib vs*. *Rb/p53* lung tumors, and TET-*Nfib* cell lines with or without DOX, we found 66 genes to be commonly changed depending on *Nfib* status (Figure 4C, Supplemental Table 5). 62 of these genes changed in the same direction, 43 were up-regulated when *Nfib* expression was increased and 19 down-regulated. KEGG pathway analysis of these consistently *Nfib*-regulated 62 genes, showed the highest enrichment in axon guidance genes (*p* = 0.00004, Benjamani-Hochberg FDR *q* = 0.001). Axon guidance related genes including *Epha5, Epha8, Slit1* and *Unc5d* were upregulated and *PlexinA1* and *Robo1* downregulated upon *Nfib* overexpression (Figure 4D, Supplemental Figure 3).

## DISCUSSION

The most metastatic form of lung cancer is small cell lung carcinoma (SCLC). Unfortunately, there is little known about the signaling pathways that control SCLC metastasis. Human metastatic SCLC is rarely resected, hindering our understanding of genetic determinants of SCLC metastasis. We compared genetic differences between primary SCLC and liver metastases from genetically engineered mouse models of SCLC. We identified *Nfib* as a gene that is frequently amplified in distant liver metastases (Figure 1). *Nfib* was previously found amplified in SCLC from the *Rb/p53* model [10], but *in vivo* evidence that *Nfib* functions as an oncogene was needed. Thus, we interrogated *Nfib* oncogenic function using a sensitized mouse model of SCLC driven by inactivation of *Rb* and *p53*, genes inactivated in almost all human SCLCs. Our observation of acceleration of SCLC upon *Nfib* overexpression (Figure 2) provides definitive evidence that NFIB functions as an SCLC oncogene.

The mechanisms through which NFIB promotes SCLC are as yet unclear. We found that deletion of *NFIB* in a human SCLC cell line and NFIB suppression in cell lines derived from the *Rb/p53/TET-Nfib* SCLC mouse model led to reduced numbers of viable cells, with increased apoptosis and reduced proliferation (Figure 3). Our gene expression analysis showed that NFIB overexpression leads to activation of genes involved in axon guidance/neuronal function, which is consistent with roles for *Nfib* in brain development revealed through gene knockout studies [11]. *Nfib* also regulated genes involved in focal adhesions and extracellular matrix-receptor interactions (Figure 4). We also found reduced migration upon *NFIB* deletion in a human cell line harboring *NFIB* amplification. Our identification of NFIB-dependent gene expression differences in SCLC cells provides a starting point for functional interrogation of NFIB-regulated genes and pathways that promote SCLC.

By comparing primary SCLC to liver metastases from mouse SCLC models we found clear enrichment of *Nfib* amplifications in liver metastases (Figure 1). In matched pairs of lung tumors and liver metastases we observed *Nfib* amplifications in the metastasis but not in the bulk primary tumor. However, surprisingly, our mouse study did not reveal a higher rate of liver metastasis when *NFIB* was overexpressed at tumor initiation in the *Rb/p53* mutant model. It may be that the acceleration of primary tumor development by *Nfib* overexpression limited our ability to detect a role for *Nfib* in driving liver metastasis, since the *Nfib* overexpressing mice died earlier with less time for metastasis to develop. Transplant experiments will help us address whether NFIB overexpressing tumor cells derived from the *Rb/p53/TET-Nfib* model exhibit increased propensity to metastasis. Also using the novel *Rb/p53/TET-Nfib* mouse model described, it will be interesting to activate *Nfib* expression not at tumor initiation but later in tumor progression. Following detection of lung tumor burden by magnetic resonance imaging, *Nfib* expression could be induced and the effect of *Nfib* overexpression in tumor progression discerned. For example, numbers of circulating tumor cells and influence of *Nfib* overexpression on rate of liver metastasis in this context could be assessed. It will also be interesting to remove doxycycline from *Nfib*-driven SCLC and assess the requirement for *Nfib* in the maintenance of lung tumors and liver metastases.

In human SCLC, NFIB expression can be deregulated through multiple mechanisms. It was previously shown that 16/46 human SCLC cell lines harbored *NFIB* copy number gains, including occasional focal high-level amplifications [10]. NFIB can also be highly expressed in SCLC cells through the presence of atypically large enhancers or super-enhancers [13, 14] and NFIB is a target gene of ASCL1, a master regulator of neuroendocrine cell fate [14]. As our mouse study shows that NFIB clearly exhibits oncogenic function in SCLC, it will be critical to compare human primary tumors and metastases to determine whether human SCLC also exhibits increased *NFIB* expression in metastases. Moreover, the extent to which genetic and non-genetic changes in human SCLC controls *NFIB* expression needs to be determined.

While this work was under review, two studies were published with data also supporting roles for NFIB in promoting SCLC and driving metastasis [21, 22]. Using a different transgenic allele, the Berns group found that *NFIB* overexpression also promoted small cell lung cancer in an *Rb/p53-*deleted background [22]. One difference compared to our work is the authors observed an increased rate of liver metastasis upon *Nfib* overexpression. It is possible that differences in the level of *Nfib* overexpression between the models could have an impact on metastasis rate. Overall, findings that *Nfib* amplifications were enriched in liver metastases over lung tumors (our study, Figure 1), along with the increased metastasis observed with NFIB overexpression in [22] and promotion of metastasis in transplant studies [21] all support NFIB as an oncogene in SCLC with roles in metastasis. NFIB was found to remodel chromatin and increase DNA accessibility in SCLC cells to promote metastasis [21]. It is now critical to identify the relevant NFIB target genes and mechanisms through which NFIB promotes advanced and metastatic SCLC.

Our data reveal that *NFIB* functions as a potent oncogene in SCLC. Patients with SCLC face a dismal clinical prognosis. It is essential that we make inroads to understand the pathways through which *NFIB* and other SCLC driver genes control the steps between tumor initiation and metastatic progression.

## MATERIALS AND METHODS

### Mice

SCLC models bearing *Rb/p53* and *Rb/p53/Pten* inactivation were previously described [4, 7, 9]. To test the effect of *Nfib* overexpression *in vivo*, the *Rb/p53* floxed model was bred to mice with a *Rosa26^Lox-STOP-Lox^* reverse tetracycline controlled transactivator (*rtTA*) allele (Jackson Laboratories) and to a novel mouse strain that we generated harboring an inducible Tetracycline Responsive Element (TRE)-*Nfib* allele. Mice were maintained on a mixed genetic background. Adenoviral Cre driven by a CMV promoter was used to infect the lungs of mice via intratracheal delivery. A week after Cre delivery, mice were switched to doxycycline-containing chow (625mg/kg, Harlan) and were maintained on this feed through the duration of the experiment. Mice were euthanized when they exhibited signs of altered breathing patterns, which was typically caused by lung tumor burden.

### Construction of TET-Nfib allele

To generate the doxycycline inducible *Nfib* transgene, the mouse *Nfib* cDNA containing an amino terminal hemagglutinin (HA) tag was placed under control of the (TetO)_7_CMV minimal promoter and the 3′ untranslated sequence and polyadenylation signal from the bovine growth hormone gene was added to generate a stable mRNA as described previously [23]. The HA-tagged *Nfib* cDNA was subcloned from pCHNFI-B2 [24] as a Not1- BamH1 (blunted with T4 polymerase) fragment into pCDNA3.1Teto-CMV-zeo [25] cut with Not1 and Xba1 (blunted with T4 polymerase). The final construct, pCMVtetoNFIB2, was verified by sequencing. This plasmid was digested with Spe1/Sph1 and the 2kb fragment containing (TetO)_7_CMVpromoter driven HA-tagged *Nfib* cDNA was purified and microinjected into F/VBN mouse oocytes by the transgenic core at Cincinnati Children's Hospital Medical Center.

### Next generation sequencing

*RNAseq*: Total RNA was isolated in Trizol (Invitrogen) and RNAseq libraries generated from oligo dT purified mRNA. We used the Truseq RNA kit (Illumina) for library generation. A HiSeq 2500 was used for sequencing, generating 50 base pair single end reads. After splicing aware alignment using Tophat [26] we used EdgeR [27] to identify differentially expressed transcripts. An FDR of 0.05 was used as a cutoff for identifying differentially expressed transcripts and the DAVID Bioinformatics Resources 6.7 online tool [28] (http://david.abcc.ncifcrf.gov/) was used to identify significant differentially expressed pathways, focusing on KEGG pathway analysis. We also generated FPKM values for each RNAseq comparison using Tophat/Cuffdiff (Supplemental Tables 6,7,8) [26].

*CNV analysis:* Genomic DNA was isolated from tumors and matched tail samples. Sequencing libraries were prepared using the NEBNext DNA library preparation kit (New England Biolabs). We used an Illumina Hiseq 2500, generating 50 base pair single end reads. The Burrows Wheeler Aligner (BWA) was used to align reads to the mm9 genome. Mapped reads were placed into 100kb bins and a read depth normalized ratio of tumor to normal was visualized using IGV. We also employed CNVseq to identify regions of copy number alteration [19].

### Real-time RT-PCR

Total RNA from tumors were isolated using TRIzol (Invitrogen) following manufacturer's instructions. cDNA was synthesized using SuperScript II reverse transcriptase (ThermoFisher Scientific). Quantitative PCR was performed with SYBR Green Real-Time PCR Master mix (ThermoFisher Scientific). Quantitative expression data were acquired and analyzed with a 7900 Real-time PCR System (Applied Biosystems). Primers were designed to detect endogenous *Nifb* and *Gapdh* (as endogenous control).

NFIB cDNA forward: GGGACTAAGCCCAAGAGACC

NFIB cDNA reverse: GTCCAGTCACAAATCCTCAGC

GAPDH (endogenous control) cDNA forward: AGCTTGTCATCAACGGGAAG

GAPDH (endogenous control) cDNA reverse: TTTGATGTTAGTGGGGTCTCG

### CRISPR mediated NFIB deletion

The lentiCRISPR v2 CRISPR/Cas9 plasmid was obtained from Addgene (plasmid #52961 deposited by Feng Zhang). Guide RNA sequences were retrieved using the CRIPSR design tool at crispr.mit.edu. Sequences of primers for amplification of CRISPR-targeted genome follow:

Control forward: CACCG GTAGCGAACGTGTCCGGCGT

Control reverse: AAACACGCCGGACACGTTCGCTACC

GFP forward: CACCG GGGCGAGGAGCTGTTCACCG

GFP reverse: AAACCGGTGAACAGCTCCTCGCCCC

NFIB#1 forward: CACCGTCCGTGCAATTGCCTATACT

NFIB#1 reverse: AAACAGTATAGGCAATTGCACGGAC

NFIB#2 forward: CACCGGAGAATCGACTGCCTGCGAC

NFIB#2 reverse: AAACGTCGCAGGCAGTCGATTCTCC

### CellTiter-Glo cell proliferation assay

Experiments were performed according to the manufacturer's instructions (Promega). Briefly, 100μl of 5000 cells were seeded in white-walled 96-well plates. Plate was equilibrated at room temperature for 30 min before added CellTiter-Glo reagent at a 1:1 ratio to the cell culture volume. The plate was then mixed for 2 minutes on an orbital shaker and incubated at room temperature for another 10 minutes to stabilize the luminescent signal. Luminescence was recorded on plate reader (BioTek) at 1 second per well integration time.

### FACS analysis

Cells were resuspended in 0.6 ml PBS and fixed with 1.4 ml ice-cold 100% ethanol. Cells were treated with 0.1mg/ml RNase and 25μg/ml propidium iodide and analyzed on a flow cytometer. FACS data was analyzed with FlowJo Software.

### Cell migration assay

Briefly, 800μl of DMEM containing 10% FBS was placed in the lower chamber of 24-well 8 micron Transwells (Falcon). 200μl of 20,000 cells were placed in the upper chamber and allowed to migrate for 17 hours at 37°C. At the end of the migration assay, the filter side of the upper chamber was cleaned with a cotton swab. The filters were then fixed in 100% methanol for 5 min, stained with crystal violet staining solution (0.05% crystal violet, 1% formaldehyde, 1% methanol in PBS) for 10 minutes and rinsed with water. The filters were air-dried and the cells that have migrated through filter pores were counted. For each migration condition, the number of cells that migrated across the filters in 4X high-power fields per insert was counted. Triplicate transwells were employed for each condition.

### Histology and immunohistochemistry

We fixed lungs in formalin for 24 h before paraffin embedding. Paraffin blocks were sectioned at four-microns and stained with hematoxylin and eosin. Paraffin sections were cut and processed from xylene through a graded ethanol series (100%, 95% and 70%) to PBS. Unmasking was performed using microwave heating in sodium citrate buffer (0.01M at pH6.0). Endogenous peroxidases were blocked with 3.5% H_2_O_2_, and immunohistochemistry was performed with an overnight incubation with anti-CGRP antibody (Sigma C8198, 1:1000). Biotin-conjugated secondary antibodies (Vector Laboratories) were used at a dilution of 1/200 in blocking solution. After secondary antibody binding, detection was performed via a biotin-peroxidase complex (Vectastain ABC, Vector Laboratories) with DAB substrate (Vector Laboratories). Haematoxylin was used to counterstain.

## SUPPLEMENTARY MATERIAL FIGURES




