# Supplementary material for: NFIB overexpression cooperates with Rb/p53 deletion to promote small cell lung cancer
Source: Oncotarget. 2016 Aug 24;7(36):57514–24. doi: 10.18632/oncotarget.11583 (PMC5295369; doi:10.18632/oncotarget.11583)
Supplement: Supplementary file 1 [file oncotarget-07-57514-s001.pdf]

NFIB overexpression cooperates with *Rb/p53* deletion to promote small cell lung cancer

Supplementary Material

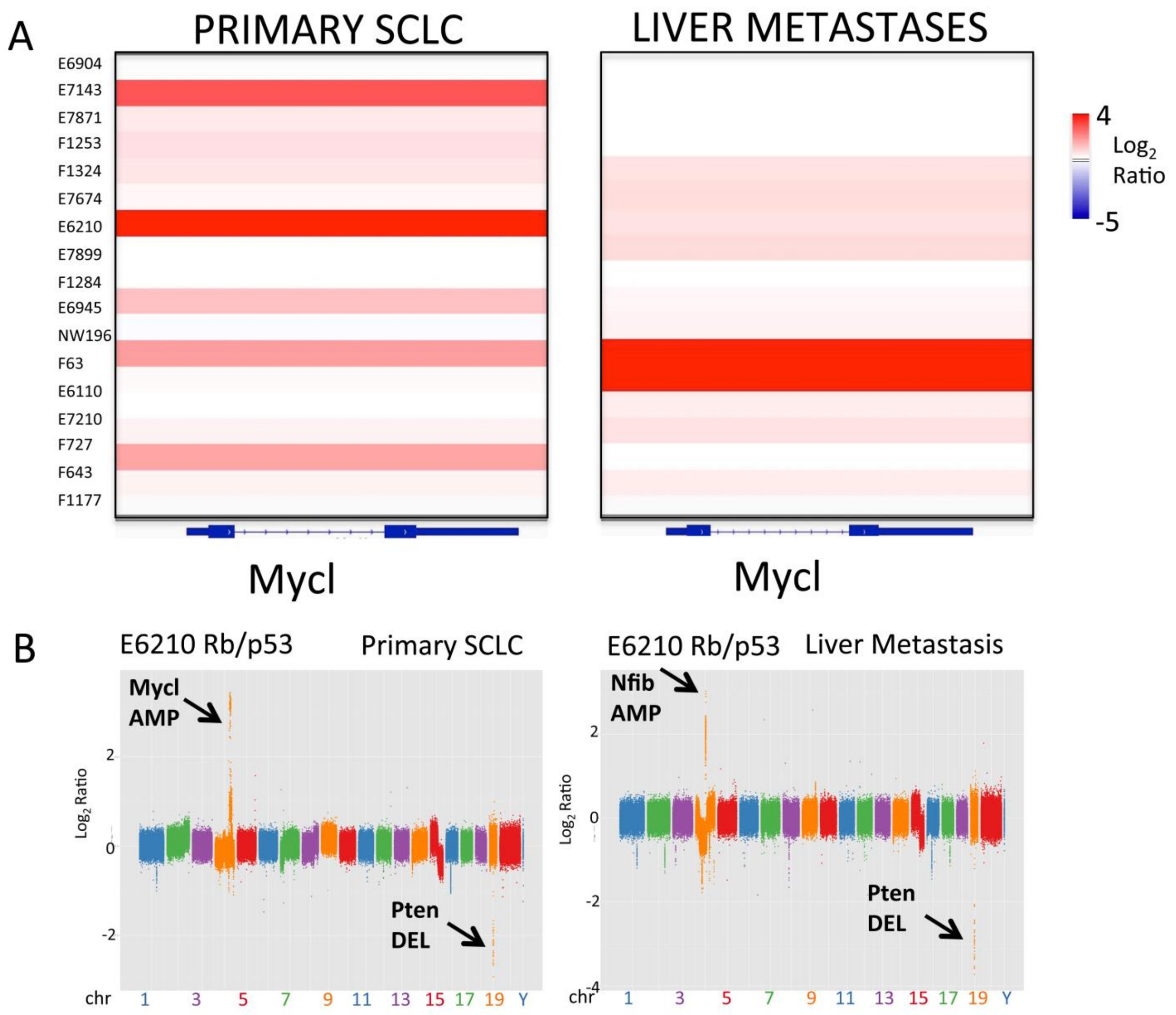

Supplementary Figure 1. *Mycl* amplifications are not enriched in liver metastases over primary SCLC. A) IGV plot showing *Mycl* copy number across 17 pairs of primary SCLC and metastatic SCLC. **B)** CNV plot from low coverage whole genome sequencing tumor pair E6210 in the *Rb/p53* model, with *Mycl* amplification in the primary tumor and *Nfib* amplification in the liver metastasis. Both harbor spontaneous *Pten* deletion (arrow).

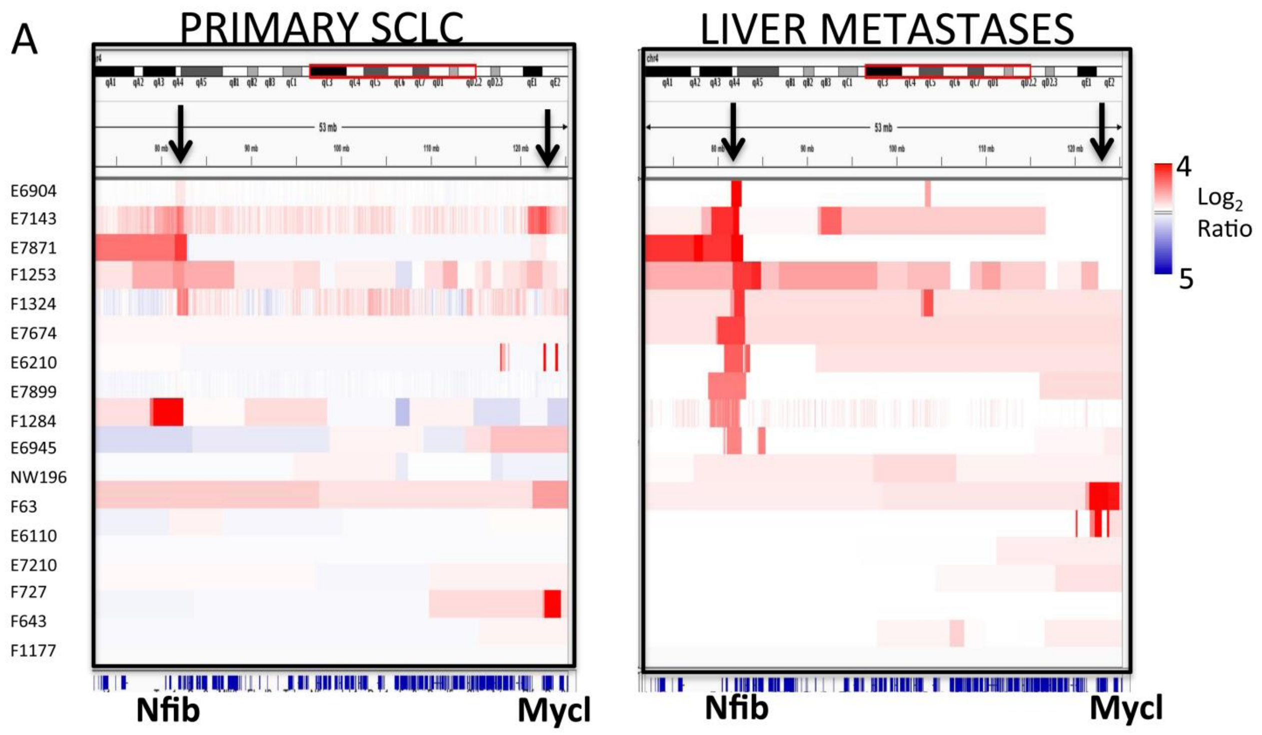

Supplementary Figure 2. *Nfib* and *Mycl* amplifications in primary and metastatic SCLC. IGV plot showing CNV changes in a region of murine chromosome 4 containing *Nfib* and *Mycl*. *Nfib* amplifications are enriched in the metastatic samples.

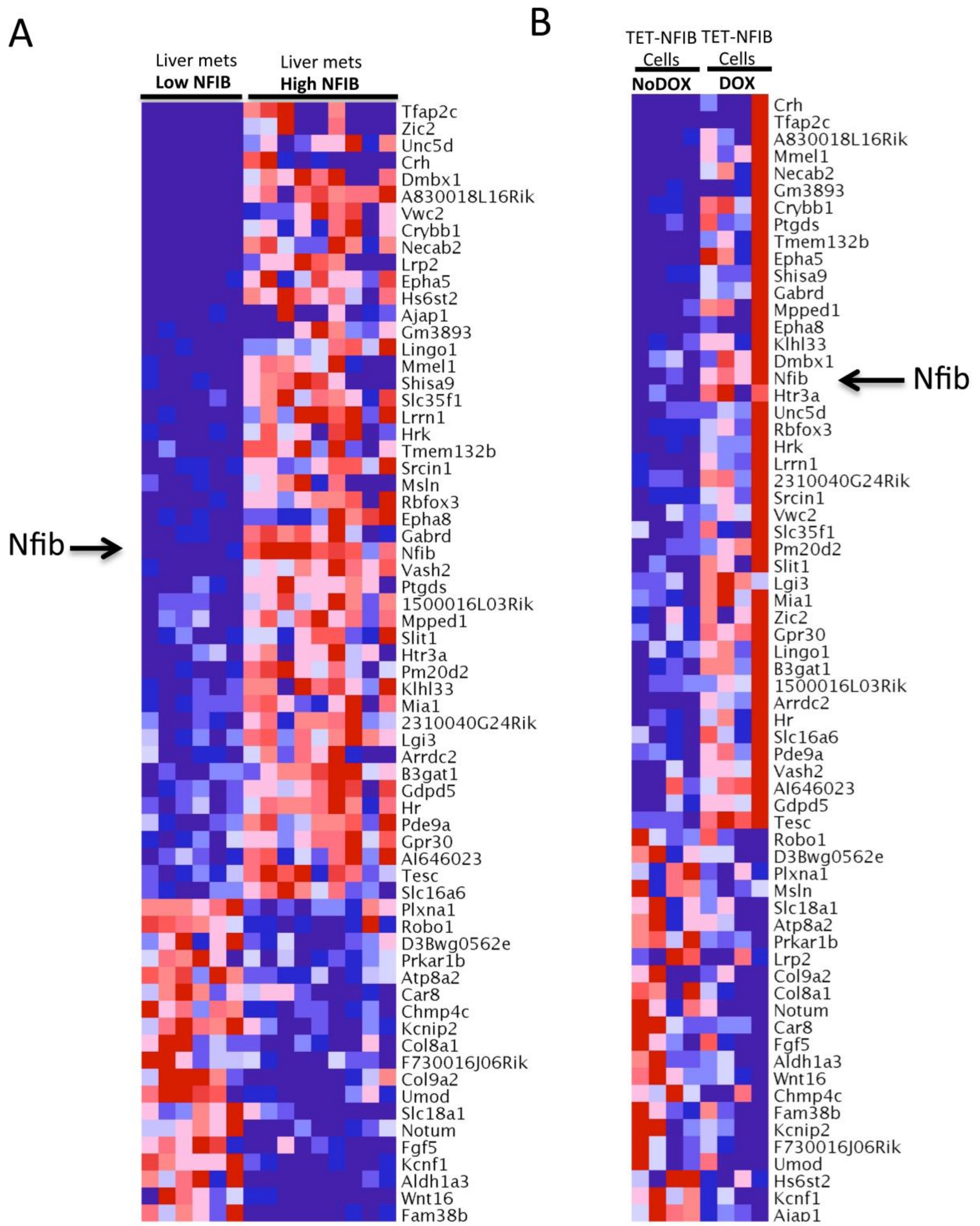

Supplementary Figure 3. Heat maps showing genes commonly differentially expressed in an Nfib-dependent manner across three independent datasets. **A)** Comparison of gene expression in liver metastases that differ in Nfib expression **B)** Comparison of cell lines derived from *Rb/p53/TET-Nfib* tumors in the presence of doxycycline (DOX) with RNA isolated 7 days after DOX removal.
